# Malaria prevalence and epidemiological characteristics in Northern Malawi: a nine-year retrospective hospital-based study

**DOI:** 10.1186/s12936-026-06046-0

**Published:** 2026-07-08

**Authors:** Qian Wu, Qi Zhang, Dokani Michael Ndovi, Shangwu Liu, Xiaoxuan He, Yanfen Ma, Jian Hu, Ying Li, Thomas Stuart Mughogho, Xiaoqin Wang

**Affiliations:** 1 Department of Clinical Laboratory, The First Affiliated Hospital of Xi’an Jiaotong University, Xi’an, 710061 Shaanxi China; 2 Laboratory Department, Mzuzu Central Hospital, Mzuzu, Malawi; 3 Department of Clinical Laboratory, Shaanxi Provincial Tuberculosis Prevention and Control Hospital, Xi’an, 710100 Shaanxi China; 4 Department of Clinical Laboratory, Shaanxi Kang Fu Hospital, Xi’an, 710065 Shaanxi China; 5 Department of Biological Sciences, Faculty of Science, Technology and Innovations, Mzuzu University, Mzuzu, Malawi

**Keywords:** Malaria, Prevalence, Epidemiology, *Plasmodium falciparum*, Parasite density, Seasonality, Malawi

## Abstract

**Background:**

Malaria remains a major public health challenge in Malawi, yet contemporary epidemiological data from northern Malawi are limited. This study aimed to characterize the prevalence, demographic distribution, temporal patterns, and parasite density of malaria using routine laboratory surveillance data from a tertiary referral hospital.

**Methods:**

A retrospective cross-sectional study was conducted using malaria screening records from Mzuzu Central Hospital, northern Malawi, collected between January 2017 and August 2025. Laboratory-confirmed malaria cases and malaria-negative individuals were included to estimate malaria prevalence across demographic and temporal subgroups. Malaria prevalence was compared using the χ^2^ test or Fisher's exact test, with Bonferroni correction for multiple comparisons. Parasite density, available for a subset of confirmed cases from 2024–2025, was summarized as median (interquartile range [IQR]) and compared using the Mann–Whitney U test or Kruskal–Wallis test.

**Results:**

A total of 31,253 individuals were screened, of whom 3405 were laboratory-confirmed malaria cases, yielding an overall prevalence of 10.89%. *Plasmodium falciparum* accounted for 86.23% of identified infections. Malaria prevalence was significantly higher among males than females (11.85% vs. 10.01%, P < 0.001). Children aged < 5 years (13.11%) and 5–14 years (13.31%) had significantly higher prevalence than adults (P < 0.001). Annual malaria prevalence ranged from 7.99 to 12.81% during 2018–2024. Clear seasonal variation was observed, with prevalence consistently peaking during the second quarter (April–June) and declining during the third quarter (July–September). Parasite density decreased significantly with age (p < 0.001), with children aged 5–14 years showing the highest median density (6,285 parasites/μL), but did not differ by gender or season.

**Conclusions:**

Malaria remains highly endemic in northern Malawi, with substantial age- and season-related heterogeneity in transmission. Children younger than 15 years experienced both the highest malaria prevalence and parasite densities, while transmission increased during and immediately after the rainy season. These findings provide updated region‑specific evidence to support targeted, age‑ and season‑tailored malaria control strategies, and highlight the need for strengthened surveillance and preventive interventions in high‑risk populations.

## Introduction

Malaria remains a major global public health challenge, causing substantial morbidity and mortality, particularly in sub-Saharan Africa [1]. The disease is caused by protozoan parasites of the genus *Plasmodium*, including *Plasmodium falciparum* (*P. falciparum*), *Plasmodium vivax* (*P. vivax*), *Plasmodium ovale* (*P. ovale*), *Plasmodium malariae* (*P. malariae*), and *Plasmodium knowlesi* (*P. knowlesi*), and is transmitted primarily through the bite of infected female *Anopheles* mosquitoes [2]. Although substantial progress has been made in malaria control over recent decades, sub-Saharan Africa continues to bear a disproportionate share of the global malaria burden, with young children, pregnant women, and non-immune individuals remaining the most vulnerable populations [3].

Malaria remains endemic in 83 countries worldwide [4]. According to the latest estimates, global malaria cases increased from 229 million in 2019 to 282 million in 2024, with the African Region accounting for approximately 94% of all malaria cases and 95% of malaria-related deaths [5–7]. Malawi is among the 15 countries with the highest malaria burden globally [6]. Despite substantial expansion of malaria control interventions over the past decade, transmission remains intense and geographically heterogeneous, with district-level incidence ranging from 155 to 1,050 cases per 1000 population in 2020 [8]. An estimated 4.3 million malaria cases were reported nationally in 2022 [9]. Malawi accounts for approximately 1.8% of global malaria cases and 1.2% of malaria-related deaths, while contributing nearly 8% of all reported malaria cases in Eastern and Southern Africa [4]. Malaria transmission in Malawi is strongly seasonal, with peak incidence occurring during and immediately after the rainy season (November–April) [10]. Collectively, these observations highlight the substantial and persistent public health and socioeconomic burden of malaria in the country.

Several studies have investigated malaria epidemiology, associated risk factors, and the effectiveness of control interventions in Malawi; however, many were conducted several years ago, focused on specific population groups, or were based on relatively small sample sizes [11–13]. Moreover, most available evidence originates from central and southern Malawi, whereas contemporary epidemiological data from northern Malawi remain limited. Long-term studies describing demographic and temporal trends in malaria transmission in this region are particularly scarce. Given the considerable geographical heterogeneity in malaria transmission driven by differences in climate, population characteristics, and access to healthcare, updated region-specific epidemiological evidence is essential for informing targeted malaria control strategies.

To address this knowledge gap, we analyzed malaria screening records from Mzuzu Central Hospital, the principal public tertiary referral hospital in northern Malawi, collected between January 2017 and August 2025. Using a large hospital-based retrospective dataset, we aimed to estimate malaria prevalence, characterize demographic and temporal patterns of malaria, and identify population groups disproportionately affected by the disease. Our findings provide updated region-specific evidence to strengthen malaria surveillance and support evidence-based prevention and control strategies in northern Malawi.

## Methods

### Study design and population

This retrospective hospital-based study analysed routinely collected data from individuals screened for malaria at Mzuzu Central Hospital between January 2017 and August 2025. Mzuzu Central Hospital is a 610-bed government tertiary referral hospital serving a population of approximately three million people in northern Malawi.

Medical records containing complete demographic information (age, gender, and date of testing) and laboratory results were eligible for inclusion. Records with incomplete demographic, clinical, or laboratory information were excluded to ensure data quality and analytical rigor.

A total of 31,253 eligible individuals were included in the final analysis. Malaria-positive and malaria-negative individuals were compared according to demographic characteristics and temporal patterns to describe the epidemiology of malaria within the hospital catchment area. In addition, parasite density among laboratory-confirmed malaria cases was analysed according to demographic and temporal characteristics.

### Blood collection and laboratory procedures

Approximately 3 mL of venous blood was collected from each participant by trained phlebotomists using standard venipuncture procedures and transferred into ethylenediaminetetraacetic acid (EDTA) vacuum tubes to prevent coagulation.

Thick and thin blood smears were prepared on clean glass slides. Thin smears were fixed with absolute methanol, whereas thick smears remained unfixed. All smears were stained with 3% Giemsa solution for 30 min and examined under oil immersion at × 1000 magnification using light microscopy.

Microscopic examination was performed to detect *Plasmodium* parasites and identify the infecting species. A blood smear was considered negative only after approximately 200 microscopic fields had been systematically examined without detecting parasites. Parasite density was quantified as the number of parasites per microlitre of blood using the standard World Health Organization (WHO) method, assuming a white blood cell count of 8,000 cells/μL.

### Data collection

Demographic and laboratory data were retrospectively extracted from the laboratory information system of Mzuzu Central Hospital. The dataset included age, gender, date of testing, malaria microscopy results, *Plasmodium* species, and parasite density where available.

Malaria prevalence was defined as the proportion of laboratory-confirmed malaria cases among all individuals screened during the study period or within each demographic or temporal subgroup.

Participants were categorized into four age groups (< 5 years, 5–14 years, 15–49 years, and ≥ 50 years) based on malaria epidemiological characteristics.

### Ethical approval

This study was conducted in accordance with the ethical principles of the Declaration of Helsinki and was approved by the Mzuzu University Research Ethics Committee (Approval No. MZUNIERC/DOR/24/166). Owing to the retrospective nature of the study and the use of routinely collected anonymized data, the requirement for informed consent was waived by the Ethics Committee.

### Statistical analyses

Statistical analyses were performed using SPSS version 30.0 (IBM Corp., Armonk, NY, USA) and R version 4.4.1 (R Foundation for Statistical Computing, Vienna, Austria). Continuous variables are presented as medians with interquartile ranges (IQR), whereas categorical variables are presented as frequencies and percentages. Malaria prevalence was calculated as the proportion of laboratory-confirmed malaria cases among all individuals screened within each demographic or temporal subgroup and is presented with corresponding 95% confidence intervals (95% CI), where appropriate. Differences in categorical variables were assessed using Pearson's χ^2^ test or Fisher's exact test, as appropriate. When overall comparisons were statistically significant, pairwise comparisons were performed using the Bonferroni correction. Temporal trends in malaria prevalence were summarized by calendar year, quarter, and month. To minimize bias resulting from incomplete annual records, data from 2017 to 2025 were excluded from temporal trend analyses. Parasite density data were non-normally distributed and are therefore presented as medians with IQR. Comparisons between two groups were performed using the Mann–Whitney U test, whereas comparisons among three or more groups were conducted using the Kruskal–Wallis test. All statistical tests were two-sided, and a P value < 0.05 was considered statistically significant.

## Results

### General characteristics of the study population

A total of 31,253 individuals who underwent malaria screening at Mzuzu Central Hospital between 2017 and 2025 were included in this retrospective analysis. The overall malaria prevalence was 10.89% (3405/31,253). Individuals with malaria were significantly younger than those without malaria (median age: 11.0 years [IQR, 6.0–25.0] vs. 17.0 years [IQR, 7.0–34.0]; P < 0.001). Overall, 15,032 (48.10%) participants were male and 16,221 (51.90%) were female.

Among the 3405 laboratory-confirmed malaria cases, *P. falciparum* was the predominant species, accounting for 86.23% (2936/3405) of infections. Other identified species included *P. malariae* (0.56%, 19/3405), *P. ovale* (0.26%, 9/3405), and *P. vivax* (0.06%, 2/3405), whereas parasite species could not be determined in 12.89% (439/3405) of positive samples (Table 1). Overall, 67.55% (2300/3405) of malaria-positive cases were diagnosed between January and June, whereas 32.45% (1105/3405) occurred between July and December, suggesting a seasonal concentration of malaria cases during the first half of the year.

**Table 1:** General information

Variables	Total (n = 31253)	Malaria negative (n = 27,848)	Malaria positive (n = 3405)	Statistic	*P*
Age (years), M (Q1, Q3)	16.00 (7.00,33.00)	17.00 (7.00,34.00)	11.00 (6.00,25.00)	Z = − 14.542	< 0.001
Gender, n (%)					
Female	16,221 (51.90)	14,597 (52.45)	1624 (47.66)	χ^2^ = 27.101	< 0.001
Male	15,032 (48.10)	13,251 (47.55)	1781 (52.34)		
Malaria Species, n (%)					
* P. falciparum*	2936 (9.39)	0 (0.00)	2936 (86.23)		

Variables	Total (n = 31,253)	Malaria Negative (n = 27,848)	Malaria Positive (n = 3405)	Statistic	*P*
* P. malariae*	19 (0.06)	0 (0.00)	19 (0.56)		
* P. ovale*	9 (0.03)	0 (0.00)	9 (0.26)		
* P. vivax*	2 (0.01)	0 (0.00)	2 (0.06)		
Undetermined species	439(1.40)	0 (0.00)	439 (12.89)		
Year, n (%)					
2017	770 (2.46)	720 (2.59)	50 (1.47)	χ^2^ = 195.886	< 0.001
2018	3734 (11.95)	3375 (12.12)	359 (10.55)		
2019	3818 (12.22)	3514 (12.62)	304 (8.93)		

Variables	Total (n = 31,253)	Malaria Negative (n = 27,848)	Malaria Positive (n = 3405)	Statistic	*P*
2021	3590 (11.49)	3284 (11.79)	306 (8.99)		
2022	3560 (11.39)	3160 (11.35)	400 (11.75)		
2023	3961 (12.67)	3576 (12.84)	385 (11.31)		
2024	4953 (15.85)	4334 (15.56)	619 (18.19)		
2025	3004 (9.61)	2518 (9.04)	486 (14.25)		

### Malaria prevalence according to gender

**Fig. 1:**
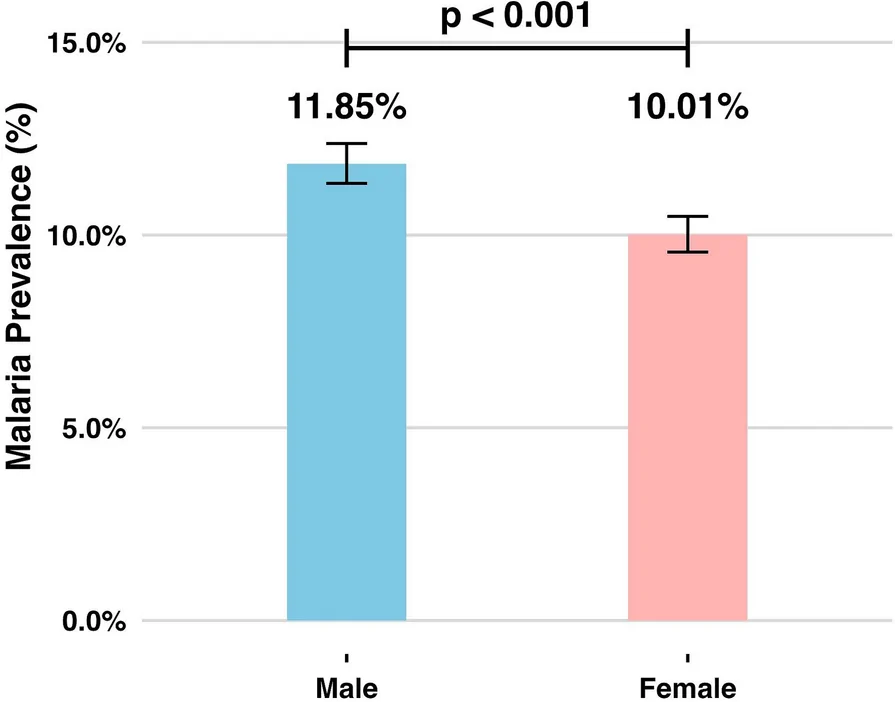
Malaria prevalence by gender

Malaria prevalence was significantly higher among males than females (11.85%, 1781/15,032 [95% CI 11.33–12.37%] vs. 10.01%, 1624/16,221 [95% CI 9.55–10.47%]; P < 0.001) (Fig. 1).

### Malaria prevalence according to age

Malaria prevalence differed significantly across age groups (χ^2^ = 148.18, P < 0.001). The highest prevalence was observed among children aged 5–14 years (13.31%, 1429/10,740), followed by children aged < 5 years (13.11%, 545/4156), adults aged 15–49 years (9.52%, 1220/12,820), and adults aged ≥ 50 years (5.97%, 211/3537).

**Fig. 2:**
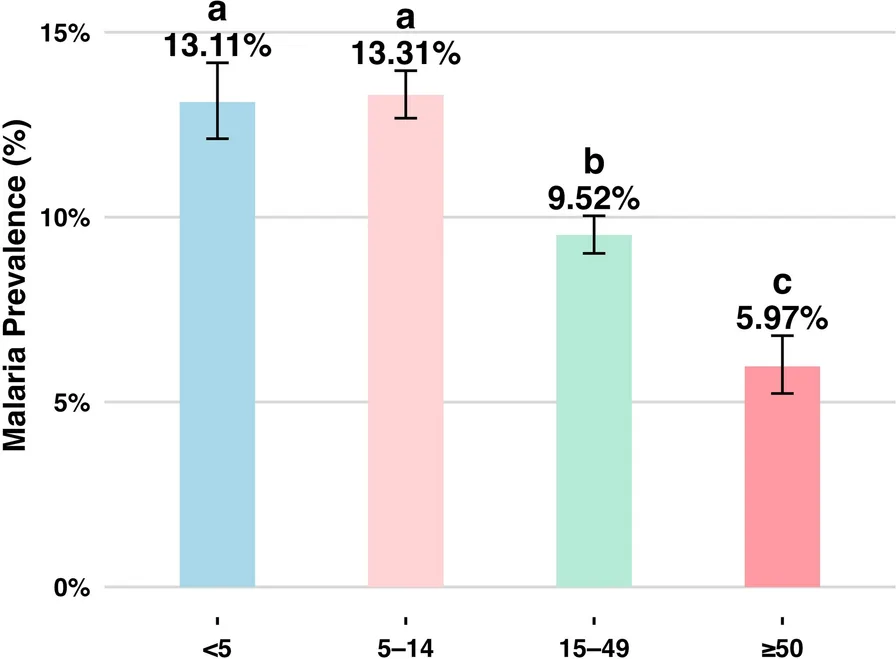
Malaria prevalence by age group

Post hoc pairwise comparisons with Bonferroni correction showed no significant difference between the two paediatric age groups (P = 0.757), whereas both groups had significantly higher prevalence than either adult age group (all P < 0.001). Malaria prevalence was also significantly lower among adults aged ≥ 50 years than among those aged 15–49 years (P < 0.001) (Fig. 2; Table 2).

**Table 2:** Age‑specific malaria prevalence, 95% confidence intervals, and pairwise comparisons among study participants

Age group	Positive cases/Total screened	Prevalence, % (95% CI)	Significance group†
< 5 years	545/4156	13.11 (12.1–14.2)	a
5–14 years	1429/10740	13.31 (12.7–14.0)	a
15–49 years	1220/12820	9.52 (9.0–10.0)	b
≥ 50 years	211/3537	6.0 (5.2–6.8)	c

^†^ Groups sharing the same letter are not significantly different (Bonferroni-corrected pairwise comparison, < 5 vs. 5–14: χ^2^ = 0.10, p = 0.757). All other pairwise comparisons were significant at p < 0.001. Overall test for heterogeneity across the four groups: χ^2^ = 198.98, p = 0.000

**Fig. 3:**
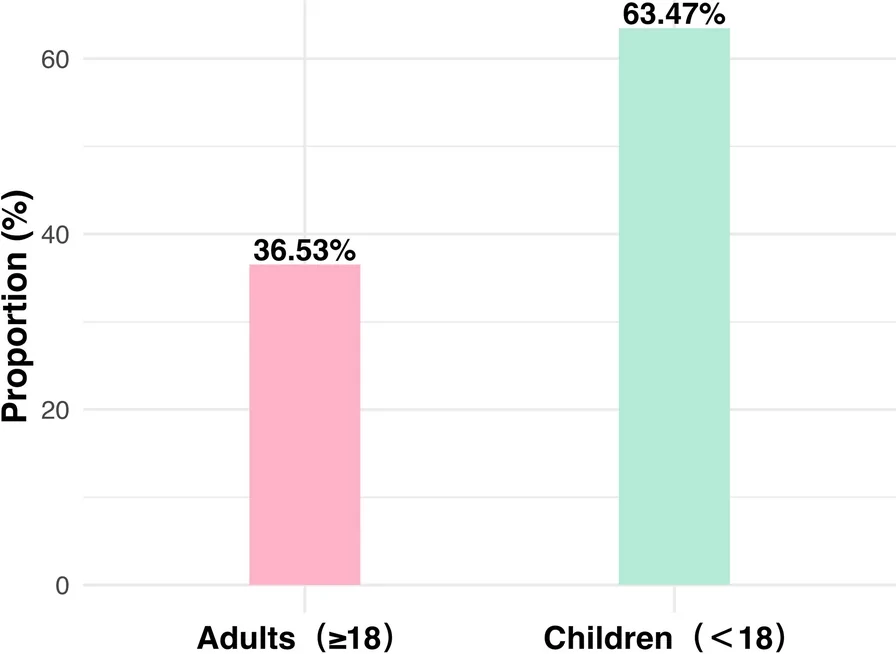
Distribution of laboratory-confirmed malaria cases by age category. Proportion of malaria-positive cases occurring among children (< 18 years) and adults (≥ 18 years). Percentages represent the distribution of confirmed malaria cases rather than malaria prevalence

Among all malaria-positive cases, 63.46% (2161/3405) occurred in children (< 18 years), whereas 36.54% (1244/3405) occurred in adults (≥ 18 years), indicating that the malaria burden was disproportionately concentrated among children (Fig. 3).

Malaria prevalence among four age groups (< 5 years, 5–14 years, 15–49 years, and ≥ 50 years). Bars represent the proportion of laboratory-confirmed malaria cases among all individuals screened within each age group. Error bars indicate 95% confidence intervals.

### Temporal and seasonal patterns of malaria prevalence

**Fig. 4:**
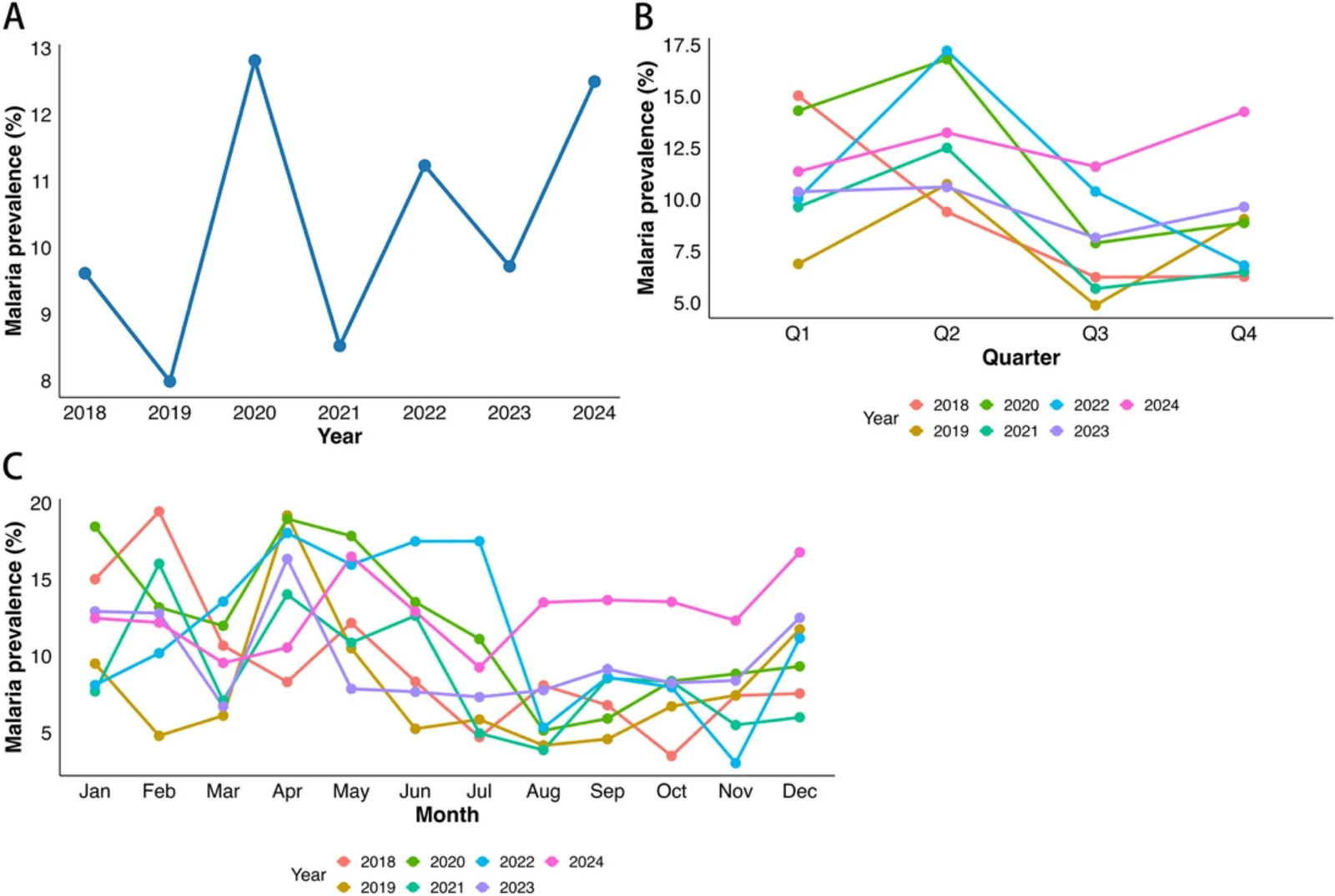
Temporal trends in malaria prevalence from 2018 to 2024. **A** Annual malaria prevalence. **B** Quarterly malaria prevalence by year. **C** Monthly malaria prevalence by year. Malaria prevalence was calculated as the proportion of laboratory-confirmed malaria cases among all individuals screened during each time period. Data from 2017 to 2025 were excluded because only partial-year data were available

To minimize bias arising from incomplete annual records, data from 2017 to 2025 were excluded from temporal trend analyses. Annual malaria prevalence ranged from 7.99% (305/3818) to 12.81% (495/3863) during 2018–2024, with the highest prevalence observed in 2020 (12.81%, 495/3863) and the lowest in 2019 (7.99%) (Fig. 4A).

Marked seasonal variation was observed across study years (Fig. 4B). In most years, malaria prevalence peaked during the second quarter (April–June), reaching 17.21% (163/947) in 2022, whereas the third quarter (July–September) generally showed the lowest prevalence. Monthly analyses demonstrated a comparable pattern, with malaria prevalence increasing during and immediately after the rainy season and declining during the dry season (Fig. 4C).

### Parasite density according to age, gender, and season

Parasite density analysis was restricted to 670 laboratory-confirmed malaria cases from 2024–2025, when parasite density was routinely recorded.

**Fig. 5:**
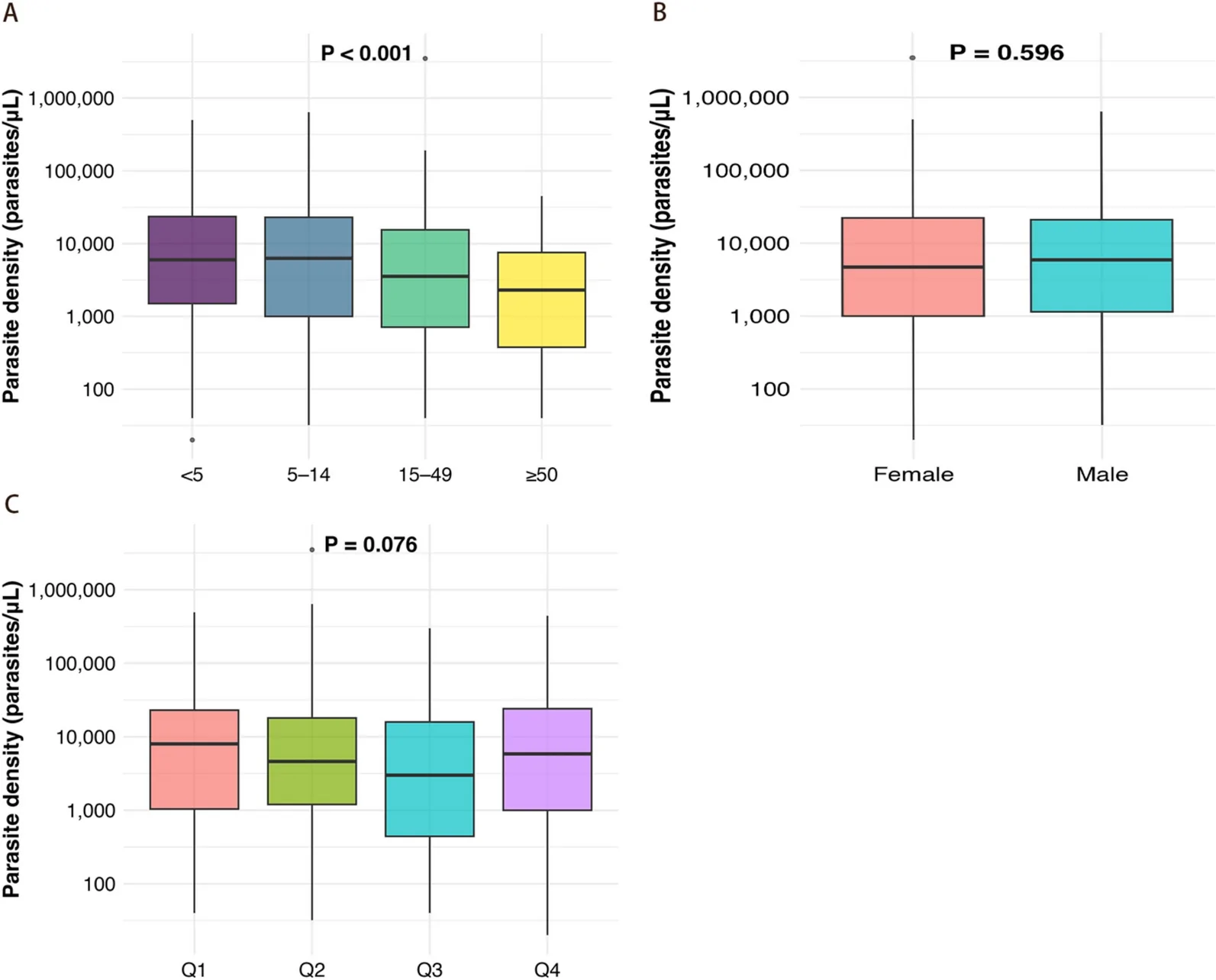
Parasite density according to age, gender, and calendar quarter. **A** Parasite density by age group. **B** Parasite density by gender. **C** Parasite density by quarter. Box plots show the median (horizontal line), interquartile range (box), and 1.5 × interquartile range (whiskers). Parasite density was expressed as parasites/μL

Parasite density differed significantly across age groups (P < 0.001) (Fig. 5A). The highest median parasite density was observed among children aged 5–14 years (6285 parasites/μL; IQR, 1,000–23,000), followed by children aged < 5 years (6000 parasites/μL; IQR, 1500–23,555), adults aged 15–49 years (3544 parasites/μL; IQR, 710–15,467), and adults aged ≥ 50 years (2300 parasites/μL; IQR, 407–7600).

No significant difference in parasite density was observed between males and females (P = 0.596). The median parasite density was 5896 parasites/μL in males and 4702 parasites/μL in females (Fig. 5B). Likewise, parasite density did not differ significantly across calendar quarters (P = 0.076) (Fig. 5C).

## Discussion

This study provides updated epidemiological evidence on malaria in northern Malawi based on a large retrospective hospital-based dataset collected over a nine-year period. By analysing more than 31,000 malaria screening records, we demonstrated that malaria transmission in the Mzuzu healthcare catchment area remains characterized by the predominance of P. falciparum, a disproportionately high burden among children, significant seasonal variation, and modest gender differences in malaria prevalence. These findings provide contemporary region-specific evidence to support malaria surveillance and guide targeted control strategies in northern Malawi.

The predominance of *P. falciparum* observed in this study is consistent with previous reports from Malawi and other malaria-endemic countries in sub-Saharan Africa, where this species accounts for the vast majority of malaria cases and malaria-related deaths [14]. This finding indicates that P. falciparum remains the principal target for malaria surveillance and control programmes in northern Malawi.

Children aged < 15 years experienced the highest malaria prevalence in our study, consistent with previous reports from Malawi and other malaria-endemic countries in sub-Saharan Africa, where children bear a disproportionate burden of malaria morbidity and mortality [4, 9]. This age-related pattern is likely explained by limited naturally acquired immunity during early childhood, whereas repeated exposure to *Plasmodium* parasites gradually leads to the development of partial protective immunity with increasing age [15, 16]. Consistent with this explanation, parasite density also differed significantly across age groups, with the highest median parasite densities observed among children younger than 15 years and progressively lower densities among adults. This finding further supports the role of age-dependent immunity in limiting parasite multiplication following infection [17, 18]. Higher parasite burdens in children may also increase the risk of symptomatic disease and contribute to ongoing malaria transmission, underscoring the importance of prioritizing early diagnosis, prompt treatment, and effective preventive interventions in this vulnerable population [9, 19].

The higher malaria prevalence observed among males is consistent with findings from Ethiopia, Nigeria, and other malaria-endemic settings [20–22]. This disparity is likely multifactorial and may reflect behavioural differences, including greater outdoor occupational exposure, increased nighttime activities, and differences in the use of personal protective measures, all of which may increase exposure to mosquito bites [23, 24]. Despite the higher prevalence among males, parasite density did not differ significantly between genders, suggesting that gender-related differences are more likely attributable to exposure than to biological differences in parasite multiplication or host immune responses. Similar findings have been reported elsewhere [25–27], although conflicting evidence exists [15, 28], indicating that further multicentre studies are warranted.

The marked seasonal variation observed in this study is consistent with the climatic characteristics of Malawi, where malaria transmission closely follows the rainy season (November–April) [29]. Malaria prevalence consistently increased during the second quarter (April–June), immediately following the rainy season, when abundant breeding sites and favourable environmental conditions promote mosquito survival and transmission. Similar seasonal patterns have been reported in Burkina Faso and other regions of sub-Saharan Africa, highlighting rainfall and temperature as major determinants of malaria transmission [30–32]. Identification of April as the peak transmission month and August as the seasonal minimum provides practical evidence for optimizing the timing of vector control interventions, including indoor residual spraying, insecticide-treated net distribution, and other seasonal prevention strategies.

The present findings have several implications for malaria control in northern Malawi. First, the substantial malaria burden among children, particularly those aged 5–14 years, suggests that current prevention programmes may insufficiently target this age group and supports expanding school-based malaria interventions. Second, the pronounced seasonal increase in malaria prevalence provides a clear opportunity for implementing intensified vector control and other preventive measures before and during the rainy season. Third, the higher malaria prevalence observed among males highlights the importance of behavioural interventions and health education aimed at reducing exposure associated with nighttime outdoor activities and occupational risks.

Several limitations should be acknowledged. As a single-centre hospital-based study, our findings may underestimate the true community burden of malaria because asymptomatic infections and cases managed at primary healthcare facilities were not captured. The retrospective study design may also have introduced selection bias. Furthermore, the absence of entomological data prevented assessment of vector species composition and seasonal vector dynamics, while information on important individual-level risk factors, including insecticide-treated net use, housing conditions, and socioeconomic status, was unavailable, limiting further investigation of determinants of malaria risk. Future multicentre studies integrating community-based surveillance with entomological investigations are needed to provide a more comprehensive understanding of malaria transmission dynamics in northern Malawi.

Despite these limitations, this study makes an important contribution to the understanding of malaria epidemiology in northern Malawi, where contemporary surveillance data remain limited. To our knowledge, it represents one of the largest laboratory-based investigations conducted in this region and provides a comprehensive analysis based on nine years of routinely collected laboratory-confirmed malaria data. The large sample size, extended observation period, and laboratory-confirmed diagnoses strengthen the robustness of the findings and provide valuable region-specific evidence on demographic and seasonal transmission patterns. These data complement national surveillance reports, address an important regional evidence gap, and establish a baseline for evaluating future malaria control interventions and informing evidence-based malaria control policies in northern Malawi.

## Conclusion

Malaria remains a major public health challenge in northern Malawi, with substantial demographic and seasonal heterogeneity in transmission. Children, particularly those younger than 15 years, bear the greatest burden of malaria, while transmission intensifies during and immediately after the rainy season. These findings provide updated epidemiological evidence from northern Malawi based on a large nine-year laboratory surveillance dataset and highlight the need for age-targeted prevention strategies, seasonally optimized malaria control interventions, and strengthened surveillance systems. The results offer valuable region-specific evidence to support malaria control planning and contribute to ongoing malaria elimination efforts in Malawi.

## Data Availability

All data generated or analysed during this study are included in this published article.
